# Bilateral Acute Osteoporotic Lumbar Pedicle Fracture Presenting with Associated Neurological Deficit: A Case Report and Review of Literature

**DOI:** 10.7759/cureus.7273

**Published:** 2020-03-14

**Authors:** James Ebot, Angela M Bohnen, Kingsley Abode-Iyamah

**Affiliations:** 1 Neurosurgery, Mayo Clinic, Jacksonville, USA

**Keywords:** pedicle fracture, compression fracture, radiculopathy, post-menopausal, osteoporosis

## Abstract

Osteoporosis is a common cause of vertebral compression fractures. Often times affecting post-menopausal women, these fractures may occur spontaneously or following minor trauma and are typically managed non-surgically. Here we present a case of a 67-year-old patient who presented with acute compression fracture of the lumbar 5 vertebra and bilateral pedicle fractures of the fourth and fifth lumbar vertebrae following an episode of coughing secondary to tracheitis. She underwent a lumbar 3 to sacral 1/ilium instrumentation/arthrodesis, with screw augmentation via hydroxyapatite, followed by lumbar 4/5 laminectomy and foraminotomy.

## Introduction

Osteoporosis is a common cause of vertebral compression fractures. Often times affecting post-menopausal women, these fractures may occur spontaneously or following minor trauma, and are typically managed non-surgically [1]. Bilateral pedicle fracture of the vertebrae is uncommon and is especially rare without trauma or high velocity activity. Even more uncommon is neurologic deficit following these fractures [2]. Here we present a case of a 67-year-old patient who presented with acute osteoporotic compression fracture of the lumbar 5 vertebra and bilateral pedicle fractures of the fourth and fifth lumbar vertebrae following an episode of coughing secondary to tracheitis.

## Case presentation

A 67-year-old female, with a history of untreated osteopenia for five years, presented to an outside emergency department with acute back pain. Her symptoms began after an episode of severe coughing related to a recently diagnosed tracheitis. She was diagnosed with a lumbar 5 compression fracture, at an outside hospital, and was managed non-surgically with medication. She subsequently presented, after one month, to our emergency room with three days of new onset bilateral lumbar 5 radicular leg pain and dorsiflexion weakness.

Physical examination revealed axial back pain with weight bearing movement and pain to percussion in the lower lumbar spine. Neurologically she demonstrated decreased sensation in the right lumbar 5 dermatome and 5/5 strength in all lower extremity muscle groups except her bilateral dorsiflexion, which was 3/5. Her body mass index was 21.

**Figure 1:**
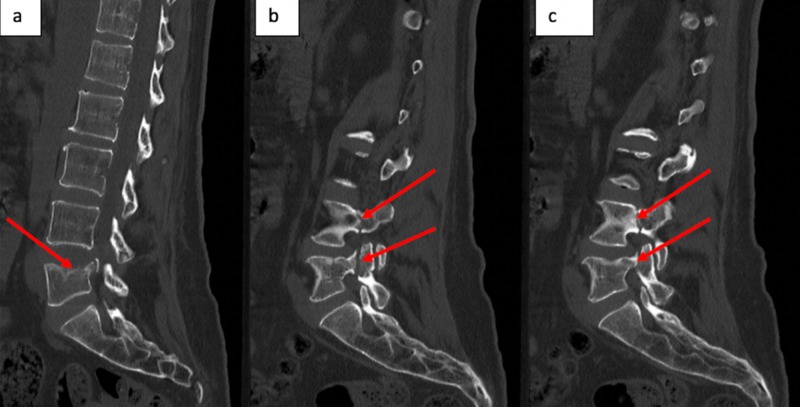
CT of the spine Non-contrast CT lumbar spine of a midline sagittal cut, which demonstrates the lumbar 5 compression fracture with retropulsed superior posterior fragment (a). Lateral sagittal images demonstrating left and right pedicle fractures at lumbar 4 and lumbar 5 (b and c).

**Figure 2:**
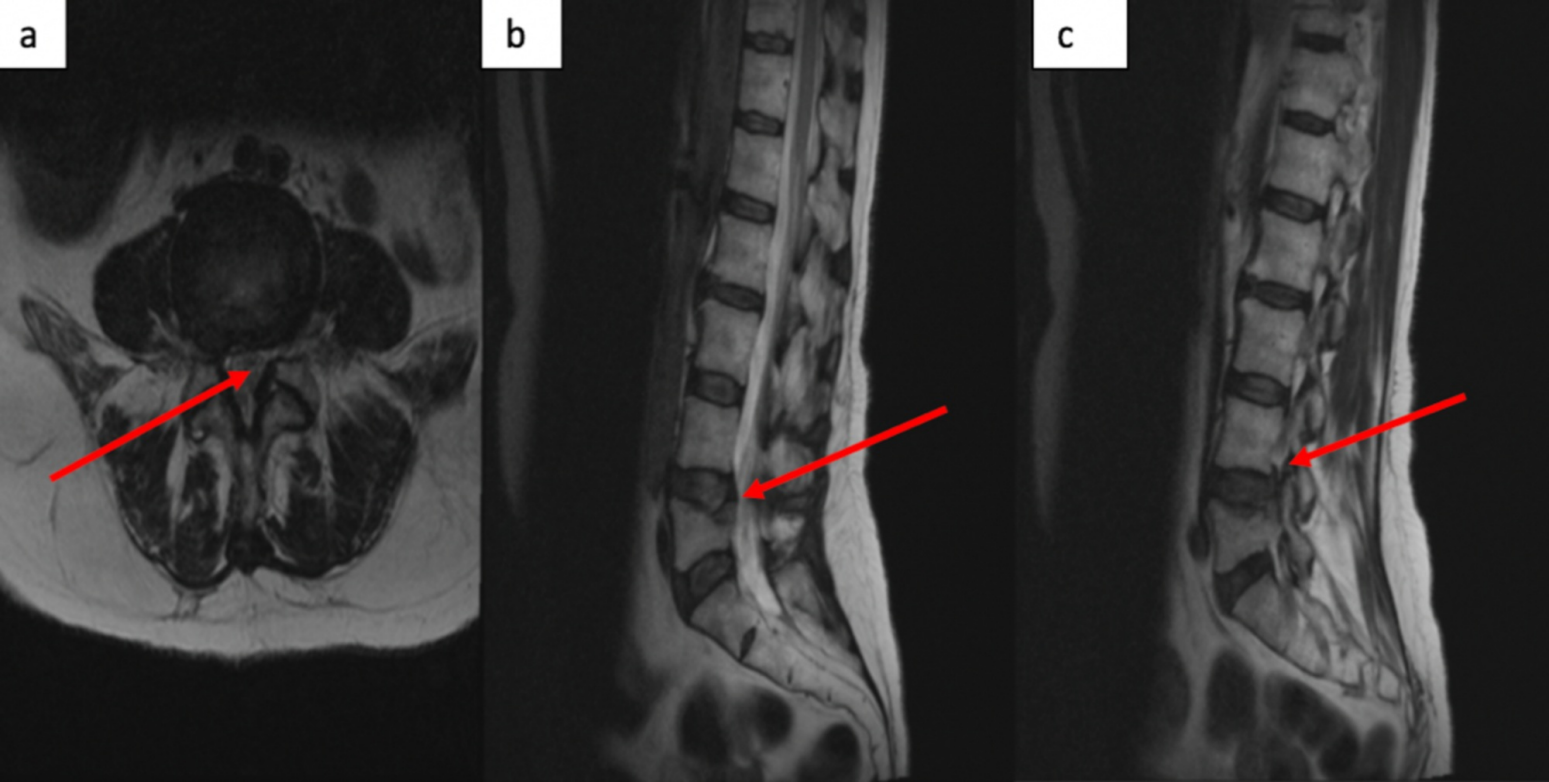
MRI of the spine T2-weighted, non-contrast MRI lumbar spine revealing central stenosis at the L4/5 level in the sagittal (b) and axial (a) views with significant thecal sac compression with severe stenosis of the traversing L5 nerve root. The lateral, sagittal view identifies moderate L4/5 foraminal stenosis with impingement on the L4 exiting nerve root (c).

CT scan of the lumbar spine revealed a lumbar 5 compression fracture as well as bilateral pedicle fractures at L4 and L5 (Figure 1). An MRI of the lumbar spine demonstrated bilateral, lateral recess stenosis causing compression of the bilateral lumbar 5 nerve roots (Figure 2).

**Figure 3:**
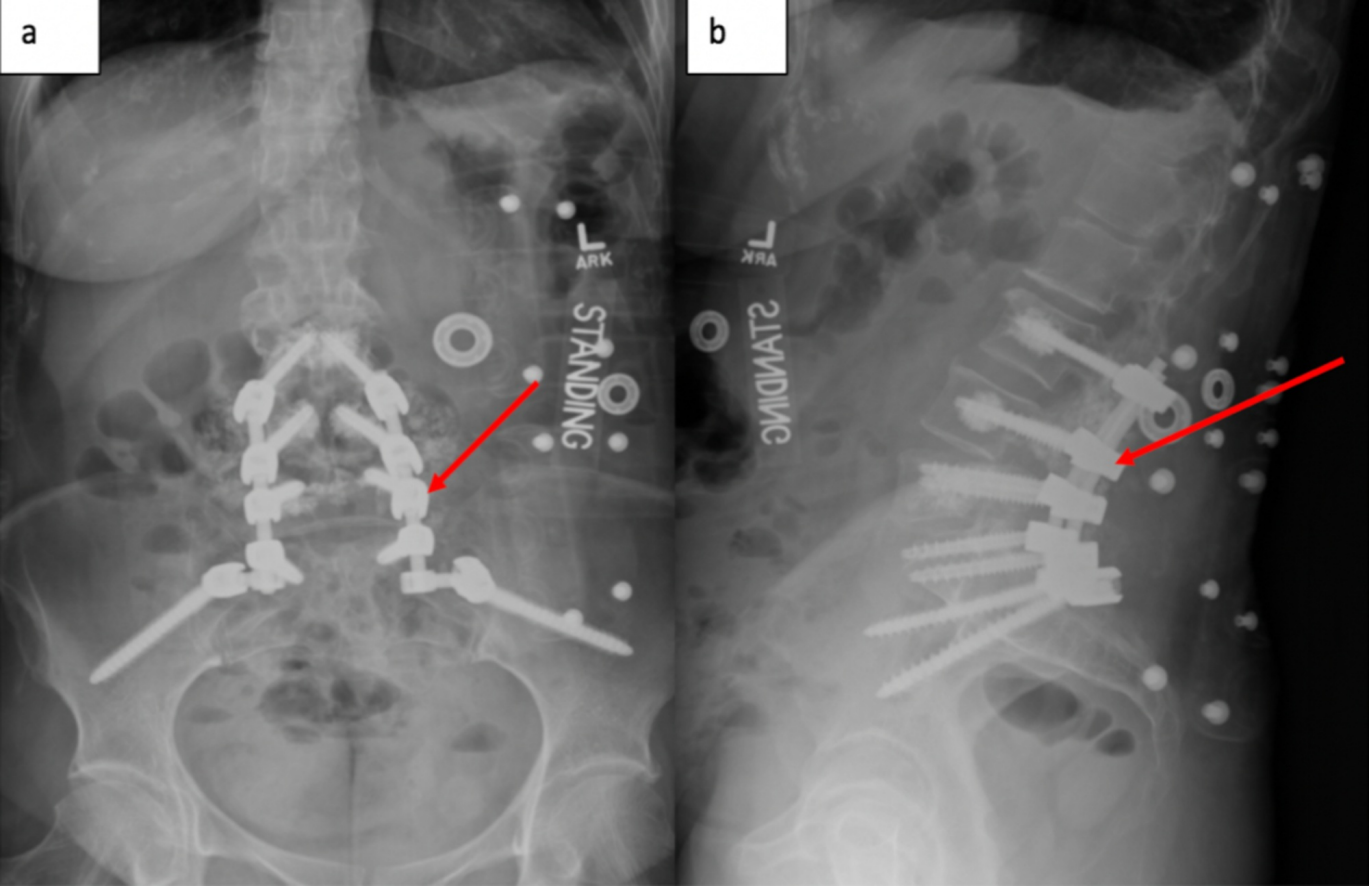
Plane films of the spine Postoperative AP (a) and sagittal (b) X-rays demonstrate the L3-S1/pelvis instrumentation. Standing - Patient is in the standing position.

Given the severity of the patient’s progressive pain and neurological deterioration, surgical intervention was discussed. She subsequently underwent a lumbar 3 to sacral 1/ilium instrumentation/arthrodesis, with screw augmentation via hydroxyapatite, followed by lumbar 4/5 laminectomy and foraminotomy (Figure 3). Intraoperative neuro-monitoring was without initial deficit or procedural changes. Postoperatively, the patient’s preoperative axial back pain significantly improved and her dorsiflexion strength improved to 5/5. The patient was subsequently discharged to rehab on day 5. On her subsequent postoperative visits, she remained pain free with 5/5 strength in her dorsiflexion.

## Discussion

As Americans age, the risk of osteoporotic fractures increases, along with their social and economic impact. It is estimated that 50% of the population 50 years and greater are at high risk for osteoporosis. Osteoporotic fractures are suspected to total more than 3 million by 2025, an increase of 48%, surpassing a national burden of $25 billion dollars in 2025 with a cumulative 10-year burden of $228 billion [3].

Vertebral body compression fractures are a frequent cause of neurosurgical consultation in the elderly population, particularly post-menopausal women [4, 5]. The most common presenting symptom is pain. Occasionally, patients complain of subjective weakness secondary to limited mobility and pain, but without objective findings. Most fractures can be treated non-surgically including pain medication, modification of activity and bracing [4]. For patients in whom mobilization is limited due to severe pain, even after bracing, a vertebroplasty or balloon kyphoplasty can be considered [5]. Surgical intervention is reserved for patients with continued fracture collapse, instability, and neural compromise.

Compression of neural elements, causing radiculopathy due to an osteoporotic compression fracture is rare [6]. Even more unlikely is the presentation of bilateral pedicle fractures in association with an osteoporotic vertebral body compression fracture [2]. Our patient had osteopenia, placing her at risk for compression fractures related to minor trauma. Her tracheitis and frequent episodes of violent coughing spells were the likely inciting event of her fractures, as her pain started immediately after a forceful episode. Our patient is unique given her fracture pattern and need for surgical intervention due to progressive neurological deterioration. In addition, our case highlights that attention to bone health and aggressive treatment of osteopenia/osteoporosis are important to prevent such fractures and minimize the morbidity that may follow.

We performed a thorough PubMed search for non-traumatic bilateral pedicle fractures. Thirteen previous reports were identified [2, 6-17] (Table 1). All patients presented with either low back pain, leg pain, or both. There were 10 females and three males, showing a female predominance. Six patients were managed conservatively and seven patients were managed surgically. Age did not seem to influence whether conservative versus surgical management was pursued. Four of the 13 patients had a known diagnosis of osteoporosis, all were females aged 50 and above.

**Table 1:** Published literature identifying non-traumatic patients presenting with bilateral pedicle fractures LBP: Low back pain; LP: Leg pain; +: Positive; -: Negative; M: Male; F: Female.

Reference	Age	Sex	Fracture	Level	Symptom	Activity / Risk Factor	Osteoporosis	Treatment
Doita et al., 2009	77	F	Bilateral pedicle	L4	LBP	Prior L5 Compression fracture	+	Surgical
Chung et al., 2002	67	F	Bilateral pedicle	L4	LP	None	+	Conservative
Kim et al., 2019	60	F	Bilateral pedicle	L2-4	LBP & LP	Unilateral L4/5 spondylosis	-	Surgical
Schmid et al., 2017	57	M	Bilateral pedicle	L4	LBP & LP	Lumbar stenosis L2-5	Unknown	Surgical
Traughber and Havlina, 1991	16	F	Bilateral pedicle	L5	LBP	Unknown	-	Conservative
Amari et al., 2009	14	M	Bilateral pedicle	L4	LBP & LP	Ballet dancer	-	Conservative
Parvataneni et al., 2004	19	F	Bilateral pedicle	L5	LBP	College lacrosse	-	Surgical
Sadiq, 2006	36	F	Bilateral pedicle	L2	LBP	Sedentary	-	Conservative
Hajjioui et al., 2011	54	F	Bilateral pedicle	L4	LBP	Unknown	+	Conservative
Ireland and Micheli, 1987	18	F	Bilateral pedicle	L2	LBP	Ballet dancer	-	Conservative
Ha and Kim, 2003	50	F	Bilateral pedicle	L5	LBP & LP	Unknown	-	Surgical
Johnson and Wang, 2009	50	F	Bilateral pedicle	L4	LBP & LP	Sedentary	+	Surgical
Doita et al., 2008	57	M	Bilateral pedicle	L4	LBP & LP	Unknown	Unknown	Surgical

Osteoporotic patients with fragility fractures require a multi-disciplinary workup, including neurosurgery, internal medicine and endocrinology. Bence Jones proteins were obtained to rule out multiple myeloma while further imaging was used to rule out the presence of any primary or metastatic spine malignancy. Once it was determined her fractures were a result of poor bone health, a decision was made to offer surgery with the goal of decompressing her neural elements and stabilizing her spine.

Minorities of postmenopausal women undergo bone health workup after an initial fragility fracture and about 50% of patients with known osteoporosis defer pharmacological treatment. Furthermore, data suggests a gap in patient education and knowledge regarding osteoporosis and bone health to be, at least, related to under-treatment [3]. Such information begs for surveillance protocols and national standards on diagnosis and treatment.

New guidelines for the diagnosis and treatment of osteoporotic fractures in post-menopausal women were published in 2019 by the International Osteoporosis Foundation (IOF) and the European Society for Clinical and Economic Evaluation of Osteoporosis and Osteoarthritis (ESCEO), aiding in algorithmic treatment of patients with increased fracture risk [18]. Practitioners should be well versed in both preventative and rebuilding factors. Anabolic treatment of osteoporosis has significant advantage in prevention of fracture risk compared to anti-resorptive medications, and plays a significant role in a subset of osteoporotic patients [19].

Post-surgically, our patient was sent to the bone health clinic for further workup and treatment. Unfortunately, as she only demonstrated osteopenia, she did not qualify for anabolic medication. Routine exercise, medication, supplementation, and surveillance will be important factors in her ongoing spine-health.

## Conclusions

We have described the unique fracture pattern and clinical presentation of a non-traumatic osteopenia-related fracture. Osteopenia/osteoporosis fractures are frequent occurrences; however, they mainly involve the vertebral body alone. Compression fractures in association with bilateral pedicle fractures are extremely rare. Moreover, neural compromise without progression of fracture is unexpected. Our case highlights the importance of heightening one’s suspicion for hidden pathology based on a patient’s nonstandard presentation. In addition, we stress the importance of a thorough, multidisciplinary approach and treatment strategy of both the fracture and underlying etiology, to maximize diagnosis and minimize comorbidity associated with the treatment of such patients.
